# Detection of metastatic tumor epithelial cells and their stemness-in lymph nodes of oral squamous cell carcinoma-a RT-LAMP pilot study

**DOI:** 10.1016/j.jobcr.2025.07.023

**Published:** 2025-08-11

**Authors:** Vandrangi Sameer kumar, M.B. Radhika, Soumya Makarla, Dipti Patil, Joyeeta Mitra, Silpa Madhuri Chikkala, Shruthi Singh, Sravya Reddy Katikela

**Affiliations:** a Dept. of Oral Pathology, Manipal University College Malaysia, Malaysia; b Department of Oral and Maxillofacial Pathology, Krishnadevaraya College of Dental Sciences, Bangalore, India; c Mahatma Gandhi Cancer Hospital, Miraj, Maharashtra, India; d Amity Institute of Biotechnology, Amity University, Uttar Pradesh, Noida, India; e Dept. of Prosthodontics, Manipal University College Malaysia, Malaysia; f Dr. D. Y. Patil Dental College & Hospital, Pune, India; g Bangalore, India

## 1 Introduction

A collection of cancers affecting the oral cavity, throat, hypopharynx, voice box, nasal cavity, and salivary glands together make up head and neck squamous cell carcinoma (HNSCC), the seventh most prevalent cancer diagnosis globally.^1^

Over 90 % of these malignancies consist of oral squamous cell carcinomas (OSCCs), which arise from the mucosal epithelium of the oral cavity.^2^

The biological nature of oral squamous cell carcinoma (OSCC) is aggressive, leading to significant destructive pathology above the clavicle, early local lymph node metastases, and the potential for distant metastases over time, even after post-effective local therapy.^3^

When compared to node-negative disease, the existence of a single micrometastatic deposit has important implications for disease progression, recurrence, and patient survival.^4^ Patients with histologically negative neck dissection specimens have been reported to have regional recurrence rates of about 10 %, indicating the presence of occult metastasis in resected nodes that went undiscovered.^5^ These metastatic deposits are small enough to evade detection by clinical-radiographic examination and conventional light microscopy. Occult metastases consist of micrometastasis, tumors deposits measuring 0.2–2 mm in diameter and isolated tumor Cells (single tumor cells or small clusters of cells not more than 0.2 mm in greatest dimension.^4^

Based on the type of neck dissection undertaken, numerous sections of lymph nodes must be analyzed, placing a significant burden on the pathologist and technician.^6^ Furthermore, pathological assessment is subjective as it is largely dependent on the person preparing the slides and the examiner.^7^ The histopathological examination of neck dissection specimens often involves analyzing multiple 3–4 μm sections from each lymph node. Micro metastases, which are tumor deposits measuring less than 2 μm in diameter, can often go undetected using conventional light microscopy.^8^^,^ ^9^

Reverse Transcription-Loop mediated isothermal amplification (RT-LAMP) is an isothermal procedure in which 6 primers are specifically designed to ensure that the target molecule is precisely identified without amplification of pseudogenes or genomic DNA. This assay is used to detect the presence of cytokeratin 19 mRNA and thus the micrometastasis.^7^

NANOG is the human ortholog of the mouse Nanog gene 1 and is a member of the homeobox family of DNA binding transcription factors. Oct 4, Sox 2, and NANOG together constitute the core transcriptional network of pluripotency. These genes collectively regulate self-renewal as well as the early stages of cellular differentiation both in vivo and in vitro.^10^

NANOG is a transcription factor that plays an important role in the maintenance of pluripotency and self-renewal in human embryonic stem cells. NANOG is a crucial factor that grants cancer cells stem-like properties such as self-renewal, tumorigenicity, metastasis, and drug-resistance.^10^ Therefore, NANOG, which promotes stem cell-like phenotype in tumor cells, is quantified using Real Time Polymerase Chain Reaction (RT-PCR) and analyzed.

## 2 Methodology

The present study, which adhered to the institutional and ethical guidelines pertaining to research involving tissue specimens, was approved by the Institutional Ethical Committee.

In the present study, patients whose primary lesion was diagnosed histologically as OSCC and clinically staged between Stage I to Stage IV (according to TNM Staging) were included. Complete detailing of patient, primary malignancy, staging, and clinical nodal status was tabulated (Table 1). A total of 32 lymph nodes excised from 7 patients were harvested from Krishnadevaraya College of Dental Sciences, Bangalore and Mahatma Gandhi Cancer Hospital, Maharashtra between October 2016 and January 2017. Each lymph node was cut into two halves:

a) One half of the Lymph node was stored in formalin and subsequently analyzed after routine Haematoxylin and Eosin (H & E) staining (gold standard).
b) The second half of lymph node was stored at −30 °C to detect Cytokeratin 19 using RT-LAMP.
c) The samples that tested positive by RT-LAMP were further processed by RT-PCR for NANOG quantification.

**Table 1:**
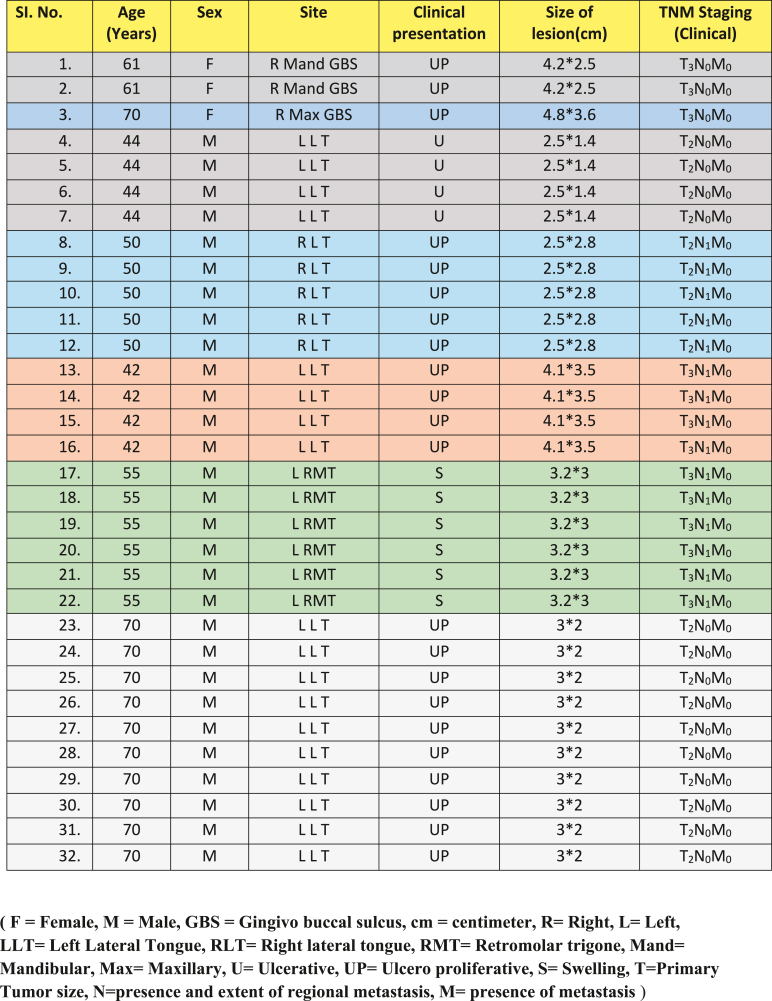
Characteristics of patients with OSCC (Pre-operative).

**Fig. 1:**
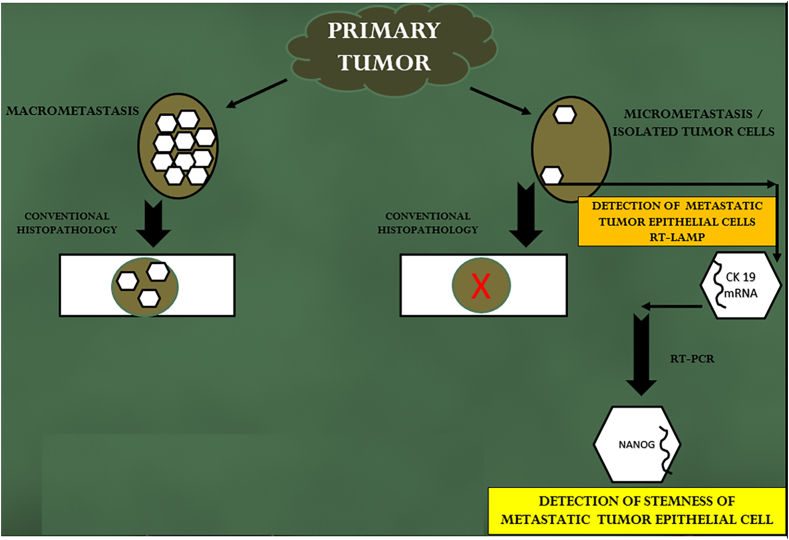
Schematic outline of the entire study design.

A schematic outline of the entire study design was depicted in Fig. 1, which clearly outlined the study workflow and highlighted the key steps of the research.

McNemar test was applied to compare RT-LAMP and conventional H & E observations. Statistical analysis was performed with the help of SPSS Software.

### 2.1 Histopathologic examination

The first half of the lymph node was subjected to step serial sectioning into multiple 100-μm slices. The sections were stained by conventional H & E staining and examined under microscope to visualize dysplastic cells or tumor cells within the lymph node parenchyma or sinusoidal spaces. The lymph nodes were categorized as positive or negative for metastasis based on the detection of tumor cells. The nodes were graded according to James S Lewis et al. for extracapsular extension.^11^

### 2.2 RT-LAMP assay

RT-LAMP was performed to detect micro-metastasis in lymph nodes by detecting Cytokeratin 19 (CK19) mRNA. This method allows for rapid and sensitive detection of metastatic cells in given samples.^7^

**Sample Preparation:** Lymph node samples stored at −30 °C were pulverized in liquid nitrogen using a sterile mortar and pestle.

### 2.3 RNA Isolation

1. **RNA Extraction:** 1 ml of RNA iso Plus Reagent (Takara Bio Inc, Japan) was added to the pulverized sample, the mixture was centrifuged at 10,000 rpm for 5 min and the supernatant was transferred into a new sterilized microfuge tube to avoid contamination.
2. **DNA Removal:** The RNA sample was treated with 1 μL of DNase I (BioLabs, New England) to eliminate any potential DNA contamination, the sample was incubated at 37 °C for 1 h and DNase was inactivated by heating the sample at 70 °C for 5 min.
3. **Spectrophotometric Analysis:** RNA purity was evaluated using a spectrophotometer (Sartorius). 1 μL of RNA was dispensed onto the pedestal of the apparatus and absorbance was checked. An absorbance ratio at 260/280 nm of 1.8–2.2 indicates high RNA purity.^12^

### 2.4 Reverse transcription

1. **Preparation for Reverse Transcription:** 100 ng of RNA was aliquoted into individual sterile microfuge tubes. 2 μL of oligo dT (Sigma Aldrich) was added to each tube. The mixture was incubated at 65 °C for 5 min, and the samples were immediately placed on ice.
2. Reverse Transcription Reaction:

A 25 μL reaction mixture was prepared containing 100 ng RNA, 2 μL of dNTPs, 1 μL of Reverse Transcriptase enzyme (BioLabs, New England), 2.5 μL of 10x Reverse Transcriptase buffer, RNase-free water. This was incubated at 42 °C for 90 min to synthesize cDNA. The reaction was terminated by heating the mixture to 70 °C for 15 min.^13^

4. **Primer synthesis and validation:** The construction of the human CK19 primers involved the creation of amplicons that spanned the exon junction regions connecting CK19 exons 1 and 2. The 6 primer sequences are
  1) 5°- TTCTCAATGGTGGCACCAACTACACGACCATCCA- 3° (CK19-FA),
  2) 5°- TCCTGCAGAGCCTCCGTCTCAAACTTGGTTCG-3° (CK19-F3),
  3) 5°- TGGTACCAGAAGCAGGGG-3° (CK19-RA),
  4) 5°- GTTGATGTCGGCCTCCACG-3° (CK19-R3),
  5) 5°- AGAATCTTGTCCCGCAGG-3°(CK19-LPF),
  6) 5°- CGTCTGGCTGCAGATGA-3° (CK19-LPR).^14^
5. **Gradient PCR for Optimal Temperature:** A gradient PCR was performed across a range of temperatures (55 °C–70 °C) using the designed primers. The PCR products were analyzed by agarose gel electrophoresis. The optimal annealing temperature was determined to be 63 °C, based on the most specific and intense amplification without non-specific bands.
6. **RT-LAMP Reaction Setup:** RT-LAMP reaction mixture was prepared containing 1 μL of cDNA template, 12.5 μL of WarmStart® LAMP 2X Master Mix (New England BioLabs), 1.6 μM each of FA and RA primers, 0.2 μM each of F3 and R3 primers, 0.4 μM each of LPF and LPR primers and RNase-free water to complete the volume to 25 μL.
7. **RT-LAMP Thermal Cycling Conditions:** The reaction was Incubated at 63 °C for 60 min. Subsequently the reaction was terminated by heating at 80 °C for 10 min to inactivate the enzymes.^12^
8. **Detection and Analysis:** amplification was detected by visual inspection by running the product on an agarose gel electrophoresis. The smeared appearance of multiple bands indicates a positive result by RT-LAMP.

### 2.5 Real Time PCR assay

The samples which were positive by RT-LAMP were further processed by RT -PCR for NANOG quantification.

The primer sequences are.

1) 5°- ATTCAGGACAGCCCTGATTCTTC-3° (NANOG-F),
2) TTTTTGCGACACTCTTCTCTGC (NANOG-R).

**PCR Reaction Setup:** Applied Biosystems Step One Real-Time PCR system was used for the analysis. The reaction mixture for each sample was prepared including 2 μL of cDNA template from the RT-LAMP positive sample, 10 μL of 2x SYBR Green PCR Master Mix (Applied Biosystems), 0.5 μL of each primer (forward and reverse, 10 μM stock), 7 μL of nuclease-free water to make up the total volume to 20 μL.

**Thermal Cycling Conditions for Real-Time PCR:** Initial denaturation of reaction mixture was performed at 95 °C for 10 min, followed by 40 cycles of denaturation at 95 °C for 15 s and annealing and extension at 60 °C for 1 min.^15^

**Data Collection and Analysis:** Fluorescence data was collected at the end of each cycle to monitor the amplification of the target gene. Data was analyzed using software to determine the Ct values for NANOG expression.

Appropriate controls were used to ensure the accuracy and reliability of the results.

## 3 Results

### 3.1 Histopathological analysis

**Fig. 2:**
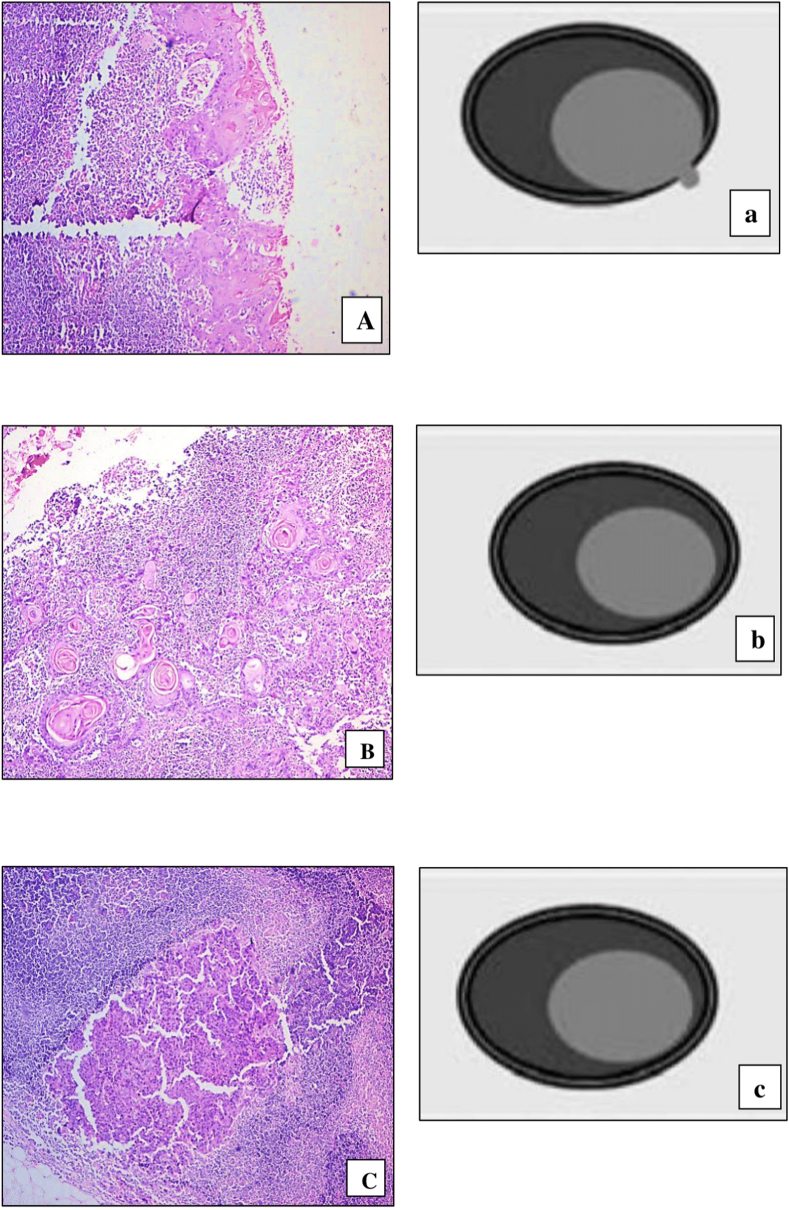
(A) Photomicrograph of Lymph node showing tumor in peri nodal tissue but extending lesser than 1mm beyond lymph node capsule (H&E stain, ×200), (a) Schematic illustration of same - Grade 2 Extracapsular Extension (ECE). (B) Photomicrograph of Lymph node showing tumor confined to the substance of lymph node. Surrounded by lymphoid tissue (H&E stain, ×200), (b) Schematic illustration of same (Grade 0 ECE). (C) Photomicrograph of Lymph node showing tumor confined to the substance of lymph node. Surrounded by lymphoid tissue (H&E stain, ×200), (c) Schematic illustration of same. (Grade 0 ECE).

**Fig. 3:**
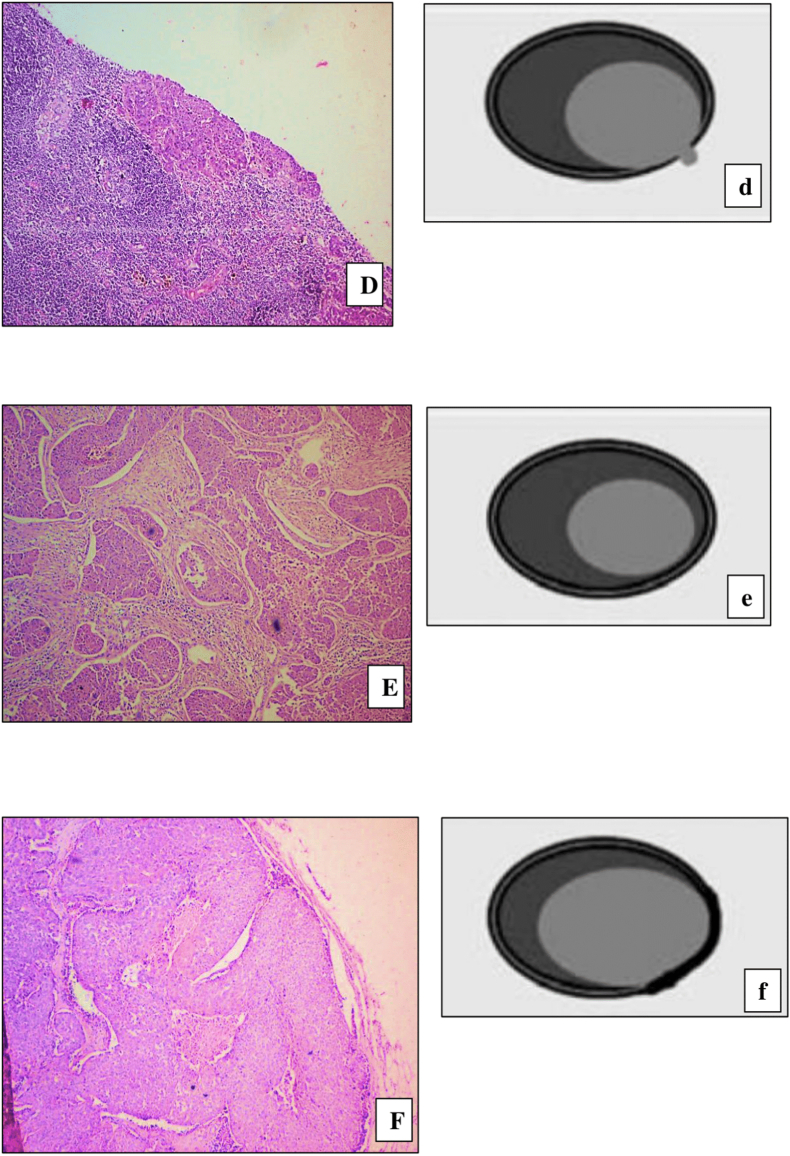
(D) Photomicrograph of Lymph node showing tumor in peri nodal tissue but extending less than 1mm beyond lymph node capsule. (H&E stain, ×200), (d) Schematic illustration of same. (Grade 2 ECE) (E) Photomicrograph of Lymph node showing tumor confined to the substance of lymph node. Surrounded by lymphoid tissue (H&E stain, ×200), (e) Schematic illustration of same. (Grade 0 ECE) (F) Photomicrograph of Lymph node showing tumor reaching the capsule of the lymph node. There is no intervening lymphoid tissue. (H&E stain, ×200), (f) Schematic illustration of same (Grade 1 ECE).

Out of 32 lymph node halves, 6 (18.75 %) nodes were found to have metastatic deposits, where 2 lymph nodes had extra capsular spread of less than 1 mm (grade II) (Fig. 2, Fig. 3 D). 1 lymph node showed tumor reaching the capsule of lymph node (Grade I) (Fig. 3 F), 3 lymph nodes showed tumor confined to the capsule (Fig. 2 B, C Fig. 3 E).

### 3.2 RT-LAMP analysis

Out of the 2nd half of all the 32 lymph nodes, a total of 15 (46.87 %) nodes showed positivity for CK 19 mRNA. i.e., along with 6 nodes which showed metastatic deposits histopathologically, 9 extra nodes showed positivity with RT-LAMP Procedure (Table 2).

**Table 2:**
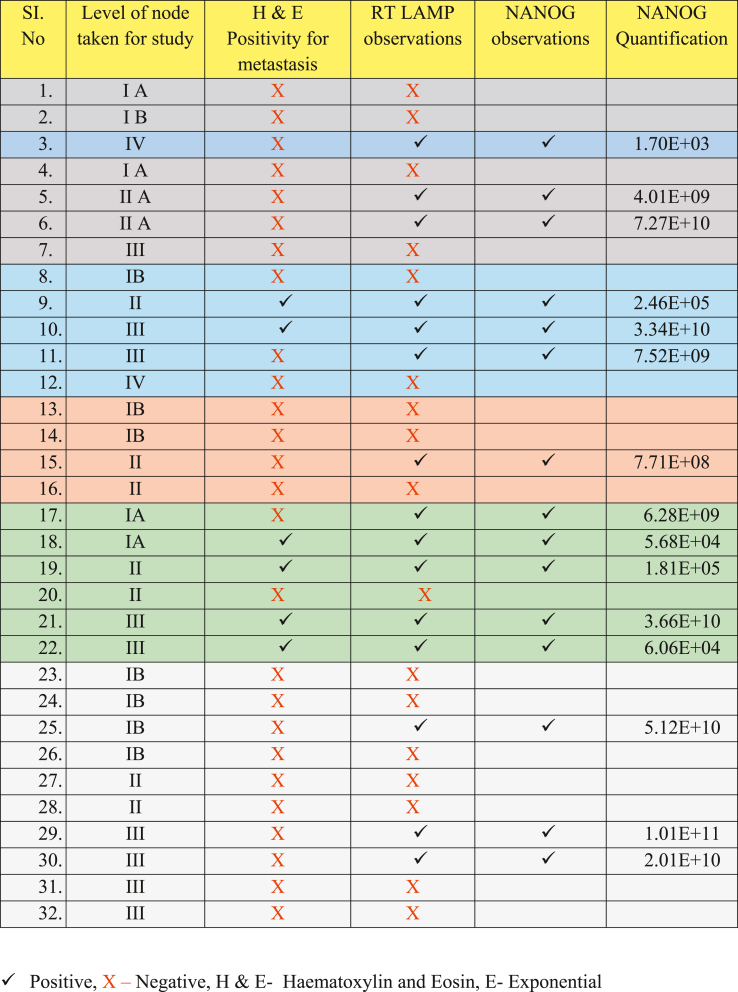
Comparison of results of lymph nodes for positivity or negativity by Histopathology and RT-LAMP technique and their corresponding NANOG observations.

**Fig. 4:**
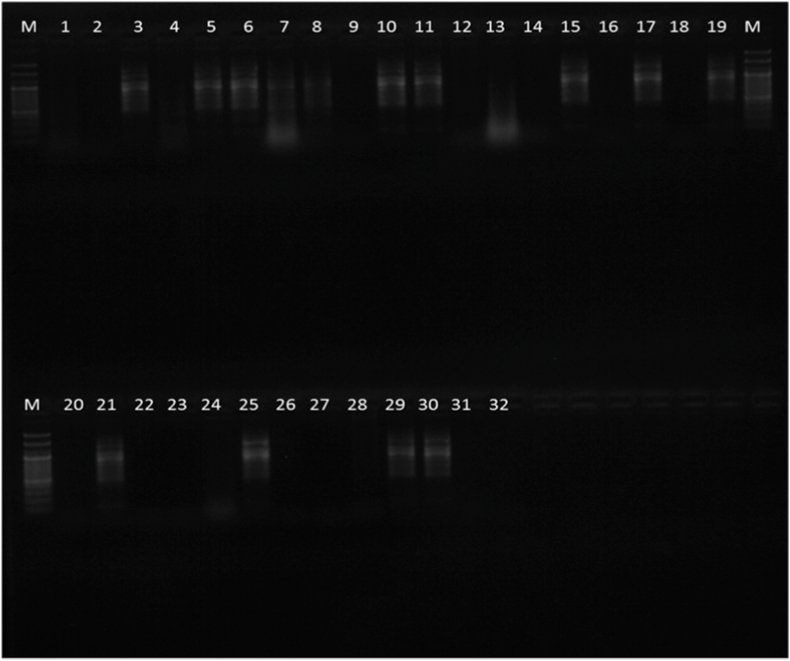
Gel electrophoresis image showing 12 samples which were positive for CK 19

**Fig. 5:**
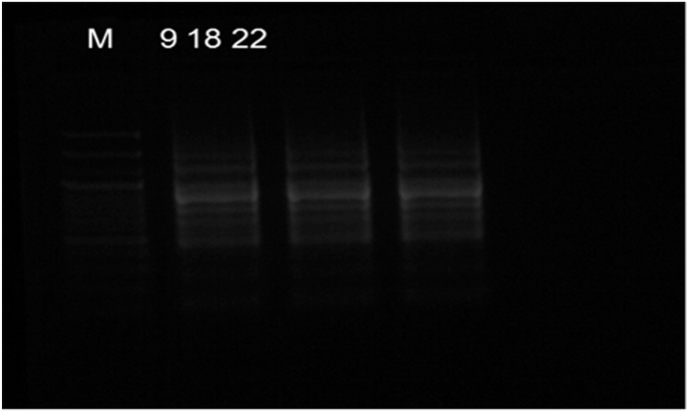
Gel electrophoresis image showing 3 samples which were positive for CK 19.

Samples 3, 5, 6, 10, 11, 15, 17, 19, 21, 25, 29 and 30 were found to have a smeared appearance of multiple bands which is characteristic of RT-LAMP technique (Fig. 3). Samples 7,8, 9, 18 and 22 (Fig. 4) appeared like bands but on repetition 7 and 8 were found to be negative and 9, 18, 22 were found to be positive (Fig. 5).

There is obvious significance of RT-LAMP technique over H & E method and the value is statistically significant. (p = 0.004).

Sensitivity of RT-LAMP is calculated to be 100 % with 95 % Confidence interval being 60.97 %, 100 %. Specificity is 65.38 % with 95 % Confidence interval being 46.22 %, 80.59 %. Diagnostic accuracy of RT-LAMP is 71.88 % with 95 % Confidence interval being 54.63 %, 84.44 %.

### 3.3 RT-PCR analysis

All the 15 nodes which were positive by RT-LAMP assay were subjected to Real Time PCR for detection of a progenitor cell marker, NANOG to predict potential stemness of these metastatic tumor cells. All the 15 samples showed significant variability, with values ranging from 1.70E+03 to 1.01E+11 (Table 2)

**Fig. 6:**
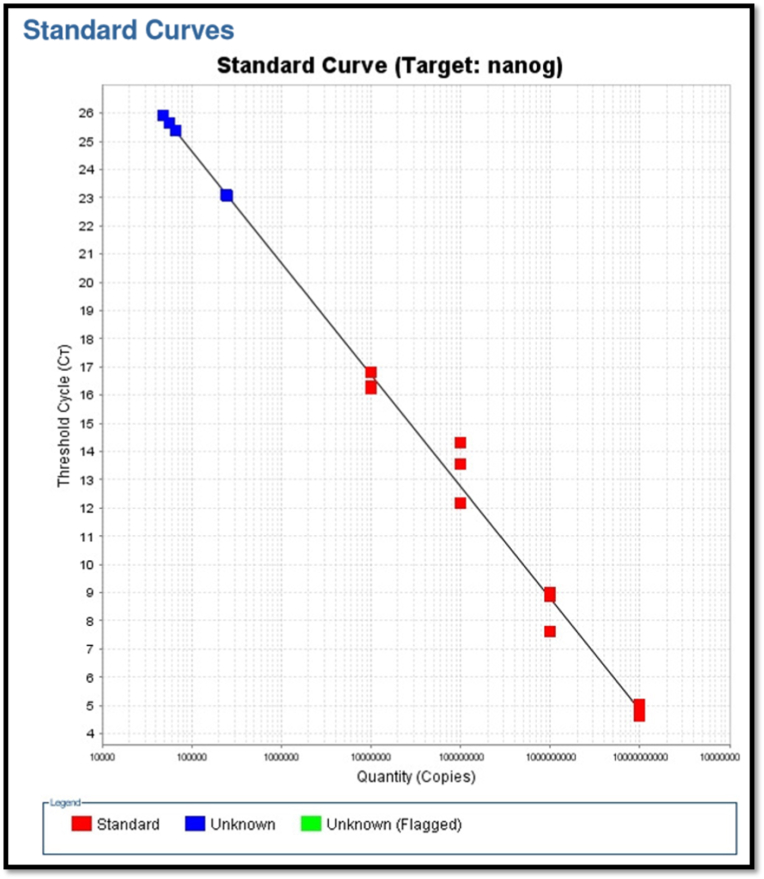
A standard curve with highest R^2^ value based on the values generated by the qPCR.

A standard curve with highest R^2^ value (Pearson Coefficient of Determination) was established based on the values generated by the qPCR and the quantity of NANOG in each sample was determined against the standard values (Fig. 6).

## 4 Discussion

Head and neck cancers (HNCs) remain a persistent and considerable global public health challenge, leading to notable mortality and illness despite substantial strides in clinical practices facilitating their early identification and management.^1^

OSCC exhibits an assertive biological demeanor and possesses a notably elevated inclination to utilize lymphatic pathways, resulting in premature lymph node metastases. Furthermore, it can even evolve into distant metastases over time, persisting despite effective local treatments.^16^

Approximately 66 % of oral squamous cell carcinomas (SCC) are already of substantial size and are accompanied by clinically observable metastases in the cervical lymph nodes upon initial diagnosis. About 8 % of patients with oral SCC will have distant metastases at the time of diagnosis, most frequently to the lungs.^16^, ^17^

Although conventional pathological assessment serves as the benchmark for identifying lymph node metastasis, it is burdened by several limitations, including instances of false negative results, variations in interpretation among different observers, an inability to thoroughly analyze entire lymph nodes, and imposing a substantial workload on laboratory personnel.^7^ So the need for a molecular technique to be used as an aid for detecting metastasis is quite evident.

Various studies have been done using conventional PCR and RT-PCR (Thakare et al., in 2013).^4^ RT-PCR is a non-isothermal procedure which makes use of a single type of primer so there are high chances of amplification of pseudogenes and genomic DNA resulting in false positive results. So there is a definite need for an advanced molecular technique.

RT-LAMP is an iso-thermal procedure, and 6 primers are specifically designed so that the target molecule is precisely identified without amplification of pseudogenes or genomic DNA. It can detect target sequences even when they are present in very small amounts. In addition, RT-LAMP offers several advantages over conventional PCR in terms of speed, simplicity, and efficiency.

RT-LAMP can amplify target DNA or RNA much faster than conventional PCR. The entire process typically takes less than an hour (approximately 40 min) compared to several hours for PCR. RT-LAMP combines reverse transcription and DNA amplification in a single step, reducing the time required for sample processing. So this is also called as One step nucleic acid amplification (OSNA).^7^

RT-LAMP is performed at a constant temperature, eliminating the need for thermal cycling required in PCR. This simplifies the equipment needed thus making it cost-effective and making it suitable for field diagnostics and point-of-care testing.^7^

As per the literature till date, various studies were done based on RT-LAMP assay. Specifically, 85 studies have focused on breast cancer, 11 on colorectal cancer, five on gastric cancer, five on lung cancer, and four on thyroid cancer. However, only two studies have been conducted to detect metastasis in cervical lymph nodes of Oral squamous cell carcinoma using the RT-LAMP technique (Hiroyuki Goda et al.^14^ in 2012, Matsuzuka et al.^18^ in 2012) and both the studies have compared efficacy of RT-LAMP technique with histopathology.

In the current study, the RT-LAMP assay has shown satisfactory effectiveness in identifying cervical lymph node metastases.

i. Higher sensitivity, 100 %, 95 % Confidence interval (CI) being 60.97 %–100 %, compared to that done by Matsuzuka et al. where sensitivity is 82.4, 95 % CI being 65.5–93.2.
ii. Comparatively lower specificity, 65.38 % with 95 % Confidence interval (CI) being 46.22 %–80.59 %: compared to that done by Matsuzuka et al. where sensitivity is 99.3, 95 % CI being 96.1–100.0.

The 2 studies that were done are quantitative whereas our study is a qualitative study which does not mention about micro or macro metastatic burden, rather it signifies presence or absence of metastasis which is very important and significant in clinical perspective and patient prognosis.

Numerous studies have conducted comparisons between RT-LAMP and histopathological examination. The findings from this research indicate that the former technique is comparable to pathological examination in detecting lymph node metastasis. However, it should be noted that achieving 100 % concordance between these studies may not be possible due to potential tumor allocation bias.^7^ On one Lymph node it is impossible to compare the efficiency of RT-LAMP over routine histopathology because of practical processing errors i.e. Lymph nodes stored in formalin cannot be used for RT-LAMP analysis.

One notable benefit of RT-LAMP in comparison to pathological examination is its capacity to objectively gather comprehensive tumor data from the entire lymph node. This is achieved with minimal or no involvement from pathologists and eliminates the inter-observer variability.

In our study, we did not intend to detect metastasis intraoperatively because of practical considerations like location of the biotechnology lab very far from the operation theatre but this method can be used for intraoperative detection of metastasis and makes One Step Surgery a realistic possibility thus avoiding a second surgery.

The presence of benign epithelial rests in lymph nodes has been seen in around 1.6 % of cervical nodes,^19^ specifically limited to lymph nodes located in level I could result in False positive results. In our study, this issue is overcome since RT-LAMP makes use of Six primers which were designed specifically so that the target CK 19 m RNA can be precisely identified without amplification of pseudogenes. Also, all the samples which showed CK 19 positivity were subjected to Real Time PCR for detection of NANOG showed positivity with variable quantification.

The lymphatic drainage of the head and neck region has a consistent and sequential pathway. Due to the presence of varied lymphatic drainage in head and neck squamous cell carcinoma (HNSCC), there is a possibility that tumor cells could go undetected in unsuspected groups of lymph nodes. The present investigation revealed such variability in lymphatic drainage patterns among a subset of patients, namely in three individuals out of a total of seven participants. Each of the three individuals exhibited primary lesions located on the tongue. Mozzillo et al.^20^ reported an incidence of 18 % in sentinel nodes that were located outside the anticipated nodal drainage pattern. Similarly, Byers et al.^21^ observed skip metastases in 15.8 % of patients diagnosed with squamous cell carcinoma of the oral tongue.

In the current study all the 15 lymph nodes showed variable degree of NANOG quantification.

Various studies using RT-PCR were carried out (Noaksson K in 2005, Collene R Jeter in 2009, Sakthi kumar Ambady et al., in 2010, Ming Li Han et al., in 2016)^22^, ^23^, ^24^, ^25^ in relation to NANOG amplification. The quantification of NANOG in the positive lymph node samples showed significant variability, with values ranging from 1.70E+03 to 1.01E+11 (Table 2) These values suggest a broad range of NANOG expression levels among the metastatic lymph nodes, indicating varying degrees of stemness within the detected tumor cells. Higher NANOG expression, as indicated by values like 1.01E+11 and 7.27E+10, reflect a higher stemness potential, which is associated with more aggressive tumor behavior and possibly poorer prognosis. In contrast, lower values such as 1.70E+03 and 5.68E+04 might suggest lower stemness potential and possibly less aggressive metastatic cells. This variability underscores the heterogeneity of tumor cell populations within the lymph nodes and highlights the need for further investigation into the clinical implications of NANOG expression levels in OSCC metastasis. As of now, there is no universal scale of quantification that could have been helpful for grading of stemness.

NANOG positivity also aids in proper documentation of inherent potential of stemness of these metastatic tumor epithelial cells and so next level of lymph nodes should be thoroughly screened and treated. This documented evidence could help in motivating the patients where most of the patients are lost in follow-up and end up in even more progressive forms of disease like distant metastasis.

## 5 Conclusion

RT-LAMP assay proves to be valuable in identifying hidden carcinomatous cells in lymph nodes of patients who have been histopathologically determined as negative.

This method is very feasible and can be used for reevaluating the status of cervical lymph node status in any type of cancer health care settings, it can be used in a corporate setup considering the rapidity of the assay and in remote areas considering the cost effectiveness of the equipment.

NANOG positivity indicates that these metastatic tumor epithelial cells had inherent potential of stemness. Thus, the surgically treated patient should be under constant follow up to screen for further spread of the cancer.

There is a need for multicenter prospective studies including a substantial number of patients to ascertain the superiority of RT-LAMP over pathological inspection in detecting micro-metastasis and individual tumor cells in cervical lymph nodes to better understand the clinical implications of NANOG expression levels in OSCC metastasis.

## Funding

This study is self-funded by me, Dr. Sameer Kumar Vandrangi.

I have done this study as a part of my thesis requirement during my post-graduation in Bangalore.

## Declaration of competing interest

The authors declare that they have no known competing financial interests or personal relationships that could have appeared to influence the work reported in this paper.
